# Occurrence of Peptide-Peptide Interactions during the Purification of Self-Assembling Peptide f1-8 from a β-Lactoglobulin Tryptic Hydrolysate

**DOI:** 10.3390/molecules26051432

**Published:** 2021-03-06

**Authors:** Mathilde Pimont-Farge, Amélie Bérubé, Véronique Perreault, Guillaume Brisson, Shyam Suwal, Yves Pouliot, Alain Doyen

**Affiliations:** 1 Department of Food Science, Institute of Nutrition and Functional Foods (INAF), Université Laval, Quebec, QC G1V 0A6, Canada; mathilde-garance-helene.pimont-farge.1@ulaval.ca (M.P.-F.); amelie.berube.3@ulaval.ca (A.B.); veronique.perreault.5@ulaval.ca (V.P.); guillaume.brisson@fsaa.ulaval.ca (G.B.); yves.pouliot@fsaa.ulaval.ca (Y.P.); 2 Arla Foods amba, 8200 Aarhus, Denmark; shykr@arlafoods.com

**Keywords:** self-assembling peptide, peptide f1-8, peptide-peptide interactions, gelation capacity

## Abstract

Self-assembling peptides have gained attention because of their nanotechnological applications. Previous work demonstrated that the self-assembling peptide f1-8 (Pf1-8) that is generated from the tryptic hydrolysis of β-lactoglobulin can form a hydrogel after several purification steps, including membrane filtration and consecutive washes. This study evaluates the impact of each processing step on peptide profile, purity, and gelation capacity of each fraction to understand the purification process of Pf1-8 and the peptide-peptide interactions involved. We showed that peptide-peptide interactions mainly occurred through electrostatic and hydrophobic interactions, influencing the fraction compositions. Indeed, the purity of Pf1-8 did not correlate with the number of wash steps. In addition to Pf1-8, two other hydrophobic peptides were identified, peptide f15-20, and peptide f41-60. The gelation observed could be induced either through peptide-peptide interactions or through self-assembling, both being driven by non-covalent bond and more specifically hydrophobic interactions.

## 1. Introduction

The enzymatic hydrolysis of whey proteins has been extensively studied to reduce the allergenicity of bovine proteins [1], as well as to generate bioactive peptides [2] and innovative ingredients with functional properties [3,4]. Of these techno-functional properties, the gelling capacity of protein hydrolysate is of particular interest for stabilizing food structures. The gelling properties are due to peptide aggregation, being linked together by non-covalent, mainly hydrophobic bonds [5,6]. Gelation can be also induced by a specific phenomenon, called self-assembly. The self-assembling properties of peptides have garnered increased interest in the last few years due to their applications in tissue regeneration, drug delivery, biosensors, and immunotherapy [7]. This process of self-assembly corresponds to the ability of a molecule to form highly ordered structures (micelles, vesicles, nanotubes, nanofibers, and hydrogels) through non-covalent interactions, such as hydrophobic and electrostatic interactions, as well as hydrogen bonding [8,9,10,11,12,13,14,15,16].

In particular, the gelation and self-assembling properties that are induced by the aggregation of peptides that are generated by whey protein hydrolysis have been well-studied over the last twenty years [5,17,18,19,20,21,22,23]. From these peptides, the peptide f1-8 (LIVTQTMK) (Pf1-8), which is generated from tryptic hydrolysis of β-lactoglobulin, is known to initiate a self-assembling phenomenon [24]. This amphiphilic self-assembling peptide has a molecular weight of 933.5 Da and an isoelectric point of 8.75 [25,26,27,28]. Its capacity to form a hydrogel is highly dependent on pH and peptide concentration, since the self-assembly is mainly controlled by hydrogen bonds, hydrophobic bonds, and electrostatic interactions [25,26,27,29].

The purification of Pf1-8 from whey protein hydrolysate is based on its insolubility in water and self-assembling properties at high concentrations [25]. Producing a purified Pf1-8 fraction requires pressure-driven ultrafiltration (UF) and reverse osmosis (RO) filtration steps, as well as consecutive wash steps [26]. However, previous studies have shown that Pf1-8 could interact with specific tryptic peptides of β-lactoglobulin, such as the peptide f15-20 (Pf15-20) and the peptide f41-60 (Pf41-60). Moreover, some studies hypothesized that Pf1-8 could precipitate and interact with non-hydrolyzed α-lactalbumin at acidic pH [29] in a β-lactoglobulin tryptic hydrolysate. Consequently, the occurrence of peptide- and protein-based interactions during the purification of Pf1-8 could interfere in the recovery and purification of this self-assembling peptide. Thus, the present study aims to understand and characterize changes in the peptide composition of the fractions recovered during production and purification of Pf1-8, especially during wash steps. This will help to evaluate the potential impact of peptide–peptide interactions on Pf1-8 purification. Finally, the gelation capacity of the different fractions recovered after each wash step was evaluated.

## 2. Results

### 2.1. Peptide Profiles of β-Lactoglobulin Tryptic Hydrolysate during the Filtration Processes

**Figure 1:**
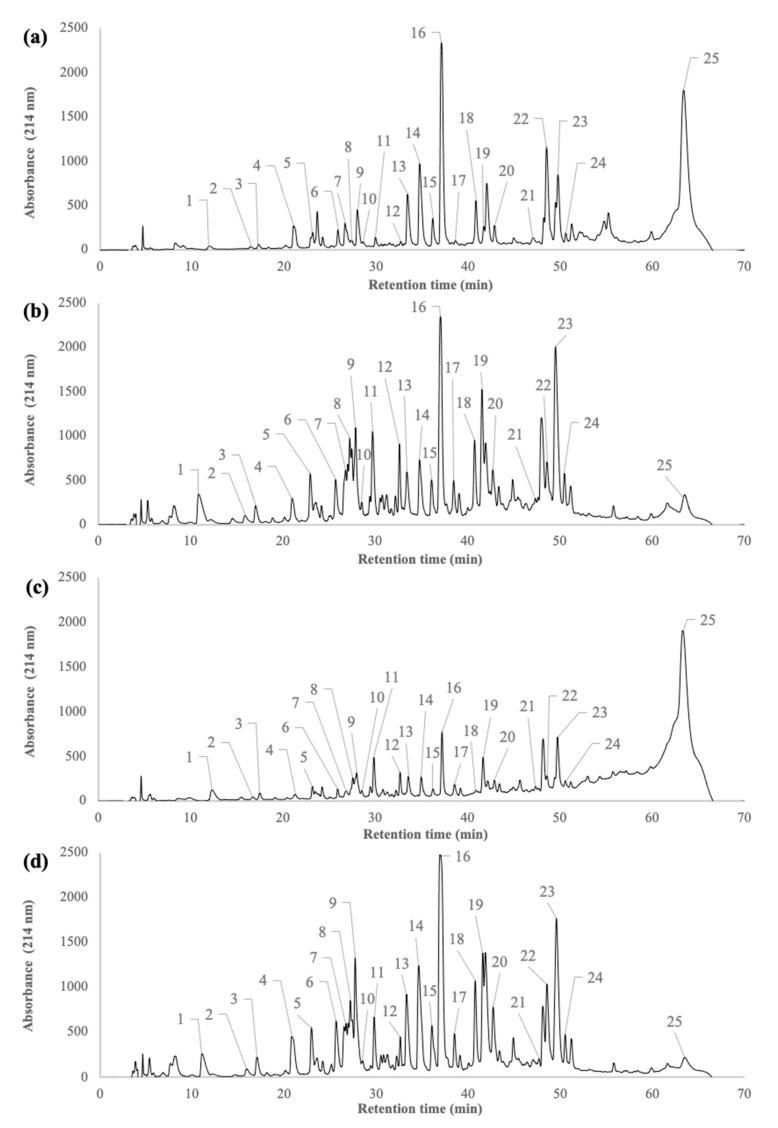
Reverse phase-high performance liquid chromatography (RP-HPLC) chromatograms of the H (**a**), the UFp (**b**), the UFr (**c**), and the ROr (**d**) after the filtration processes. Identification of peptides corresponding to the peak numbers are listed in Table 1.

Figure 1 shows the peptide profiles of the whey protein isolate (WPI) tryptic hydrolysate (H), the UF-permeate (UFp), the UF-retentate (UFr), and the RO-retentate (ROr). Table 1 reports the identification of peptides that are labelled in Figure 1, as well as their characteristics (amino acid sequence and composition, molecular weight, isoelectric point, and hydrophobicity), according to Pouliot et al. [25]. A total of 50 components were detected during the filtration and purification processes. Of these 50 components, 24 peptide sequences that were generated from tryptic hydrolysis of β-lactoglobulin and the peak corresponding to α-lactalbumin were identified in the hydrolysate, according to the peptide profiles that were reported by Pouliot et al. [25] using an identical method (Figure 1a). The remaining peptides could correspond to the hydrolysis of α-lactalbumin or other minor proteins. Specifically, the initial hydrolysate was mainly composed of peptides corresponding to peaks 9, 13, 14, 16, 18, 22, and 23 with a relative abundance that is close to 40%. After UF, all of the peptides detected in the initial hydrolysate were recovered in the UFp (Figure 1b) and the UFr (Figure 1c) but their relative abundance (peak area, not shown) was drastically different. Indeed, in the UFp, peptides 1, 5, 6, 7, 8, 9, 11, 12, 13, 14, 16, 18, 19, 20, 22, and 23 were each present at a relative abundance of more than 60%, as compared to 45% in the initial hydrolysate. In the UFr (Figure 1c), concentrations of peptides corresponding to peaks 1, 8, 11, 12, and 19 increased when compared to the initial hydrolysate. The peptide profile of the ROr (Figure 1d) was similar to that of the UFp (Figure 1b).

### 2.2. Peptide Profiles of Pellets and Supernatants Generated during the Wash and Centrifugation Steps of Pf1-8 Purification

**Figure 2:**
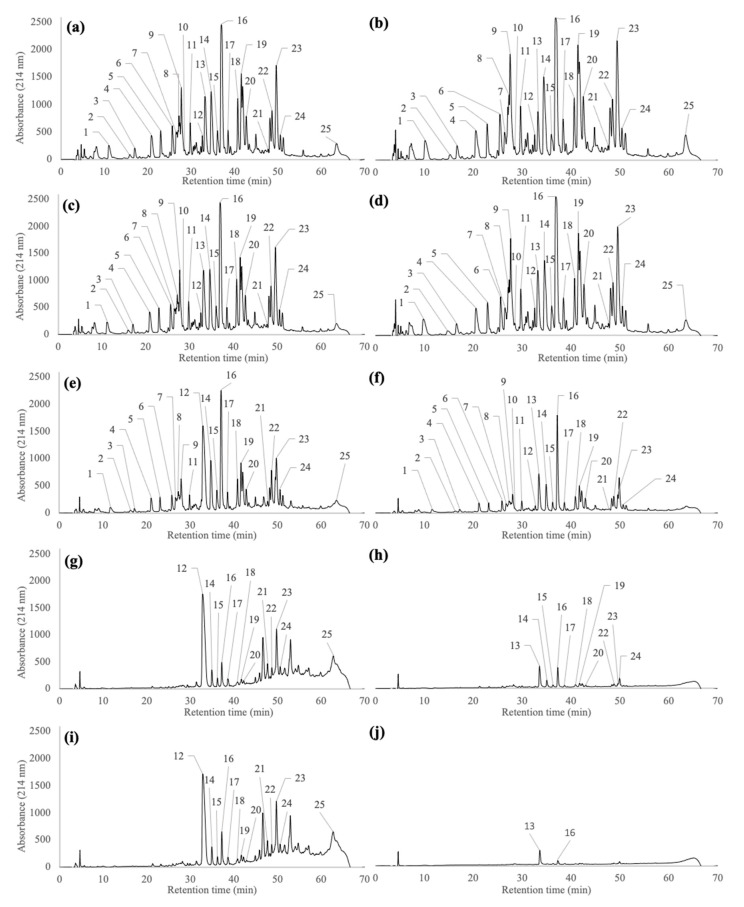
Peptide profiles of P1 (**a**), S1 (**b**), P2 (**c**), S2 (**d**), P3 (**e**), S3 (**f**), P4 (**g**), S4 (**h**), P5 (**i**), and S5 (**j**) obtained during the purification of Pf1-8 for each wash and centrifugation step. The identity of each peptide with its corresponding peak number is provided in Table 1.

Figure 2 shows the peptide profiles of the five pellets (P1 to P5) and five supernatants (S1 to S5) generated during the five wash steps of Pf1-8 purification. The total protein content (% *w*/*w*) of different pellet fractions from P1 to P5 was similar, with values close to 92%. However, important decreases in the peptide mass yield were observed after each wash step, from 12.8% after the first wash to 0.2% after the last wash, as compared to the initial weight of the peptide fraction recovered from the ROr. A total of 24 peaks associated with different peptides and one peak corresponding to α-lactalbumin were identified in P1, recovered after the first wash step (Figure 2a). The peptide/protein distribution and relative proportions of each peptide/protein (from peak 1 to 25) were similar to the results that were obtained for the ROr (Figure 1d). Almost 25 peaks were identified in S1 (Figure 2b), as observed in P1. All of the peaks identified in S1 had similar relative proportions when compared to the ROr (Figure 1d) and to P1 (Figure 2a), except for peptides 9 and 19 (which increased in relative proportion) as well as peptides 13 and 18 (which decreased in relative proportion). The peptide profile observed in P2 after the second wash step (Figure 2c) was similar to that of P1 (Figure 2a), except that the relative proportion of peptide 13 increased, and the relative proportions of peptides 9, 11, 18, 19, 20, 22, and 23 decreased. The peptide profiles of S2 (Figure 2d) were similar to S1 (Figure 2b), except that the relative proportion of peptide 6 decreased, and the relative proportion of peptide 13 increased. Moreover, the peptide profile of S2 was similar to the peptide profile of P2 (Figure 2c). Larger differences in the peptide profiles were observed after the third wash step (Figure 2e), where the relative proportion of hydrophilic peptides (1, 2, 3, 4, 5, 6, 7, 8, 9, and 11) drastically decreased compared to P1 and P2, and two peptides (10 and 13) were not recovered in P3. In addition, the relative proportion of peptide 12 considerably increased after the third wash step. The relative proportions of peptides 19 and 22 increased slightly, whereas the relative proportions of peptides 14 to 18, 20, 21, and 23 to 25 remained similar. Further, the peptide profile in S3 (Figure 2f) was significantly different from S1 and S2. The relative proportions of peptides 4, 5, 6, 7, 8, 9, 11, 12, 14, 15, 17, 18, 19, 20, 22, 23, and 24 decreased markedly. However, the relative proportions of peptides 10, 13, and 16 increased, which explains their absence, except for peptide 16, in P3 (Figure 2e). After the fourth wash step, the majority of the hydrophilic peptides from the pellets had been eliminated (Figure 2g). Peptides 1 to 11 and peptide 13 were not recovered in P4, whereas the relative proportion of peptide 12 increased. The relative proportions of peptides 21 and 23 increased slightly, while those of peptides 14 to 20, 22, 24, and 25 decreased. After the fourth wash step, 13 peptides (1 to 12 and 21), were not recovered in S4 (Figure 2h). Accordingly, the relative proportions of peptides 13, 14, 16, 19, and 24 increased in S4, whereas the relative proportions of peptides 15, 17, 18, 20, 22, and 23 decreased. Peptide 12 completely transferred to P4, whereas all of peptide 13 was recovered in S4. The peptide profile and relative proportion of each peptide in P5 (Figure 2i) were quite similar to those that were obtained in P4. The major peak that was observed in P5 (peptide 12) corresponded to Pf1-8, but some remaining hydrophobic peptides were also detected, mainly peptides 16 and 23. An increase in the purity of Pf1-8 was observed from the first to the fifth wash step. The two other main hydrophobic peptides, Pf15-20 (16) and Pf41-60 (23), were always recovered with Pf1-8 during purification. Only very low amounts of peptides 13 and 16 were recovered in S5 after the fifth wash step (Figure 2j). The last wash (S5) allowed for complete recovery of peptide 13, along with 80% of peptide 16.

### 2.3. Proportions and Concentrations of Pf1-8, Pf15-20 and Pf41-60 during the Purification Step of Pf1-8

Table 2 shows the relative proportions and concentrations of hydrophobic peptides 12 (Pf1-8), 16 (Pf15-20), and 23 (Pf41-60) in the pellet and supernatant fractions. Special emphasis was given to these peptides due to their relative abundance in fractions during the purification steps. The relative proportion of Pf41-60 was similar in both pellets and supernatants throughout the wash steps, except for S5, where it was not detected. Overall, its concentration in the different pellets remained similar at 1 mg/mL, except for the P3, P4, and P5 samples. The relative proportions (12.6 to 15.1%) and concentrations (~1.35 mg/mL) of Pf15-20 were similar in P1, P2, S1, and S2. However, after the third wash step, its relative proportion increased in S3, while remaining constant in S4 and S5, with values ranging from 20.2 to 27.1%. The last two wash steps efficiently eliminated Pf15-20 since its relative proportions decreased to 4%. The fourth wash step had a major impact on the relative proportion of this peptide between P4 and S4, as their relative proportions were 4.15% (0.381 mg/mL) and 27.1%, respectively. The last wash step was efficient, since only Pf15-20 was recovered in S5 (25.5%) and a low amount, similar to P4, was detected in P5. The purification of Pf1-8 was only observed from the third wash step, with a relative proportion of 15.4% in P3 (1.30 mg/mL). Its purification increased after the fourth wash step (35.9% in P4) and remained constant in P5 (around 3.50 mg/mL). A very low amount of Pf1-8 (<1%) was detected in S1 to 3, but, thereafter, this peptide was not detected in S4 and S5.

### 2.4. Gelation Properties of Pellet Solutions Obtained during Pf1-8 Purification

Table 3 highlights the gelation properties for pellets (P1 to P5) recovered during the purification steps of Pf1-8 by successive wash steps. Supplementary material 1 (Figure S1) shows visual observations of the gelation properties of pellets (P1 to P5). The gelation properties of P1 to P5 were tested at different concentrations, from 5 to 75 mg/mL for P1 (Figure S1a), from 5 to 20 mg/mL for P2 (Figure S1b) and P3 (Figure S1c) and at 5 and 10 mg/mL for P4 (Figure S1d) and P5 (Figure S1e). All of the pellets (P1 to P5) formed a gel, but the concentration set point varied with the number of washes. Indeed, a gel was only observed for P1 at concentrations over 40 mg/mL, for P2 and P3 at 20 mg/mL and for P4 and P5 at 10 mg/mL.

Table 4 shows the specific concentrations of Pf1-8, Pf15-20, and Pf41-60 in the peptide solutions used for the gelling tests. The concentrations of these three peptides varied in the pellet samples (P1 to P5). For all P1 concentrations tested (5–75 mg/mL), the Pf1-8 concentrations were between 0.04 and 0.64 mg/mL and they were always lower than the Pf15-20 and Pf41-60 concentrations (between 0.46 and 11.3 mg/mL). This affected gel formation, as gelation was only observed at higher concentrations (>40 mg/mL). In P1, the total relative proportion of gelling peptides (Pf1-8, Pf15-20, and Pf41-60) was around 25%, which implied that almost 75% of any solution corresponded to other peptides. Gelation for the P2 sample occurred at 20 mg/mL. Despite the fact that the Pf1-8 concentration was extremely low (<0.2 mg/mL), the concentrations of Pf15-20 and Pf41-60 were higher (>1.5 mg/mL). For P1 and P2, the ratio between Pf15-20 and Pf1-8, and between Pf41-60 and Pf1-8, were identical (20 and 10, respectively). A gel was also obtained at 20 mg/mL for P3, but with a higher concentration of Pf1-8 (close to 3 mg/mL), as observed for P2. The ratios of Pf15-20:Pf1-8 and Pf41-60:Pf1-8 in P3 were 1 and 0.4, respectively. For P4 and P5 samples, gelation was only obtained at a lower concentration of 10 mg/mL. In P4, the Pf1-8 concentration was more than 3.5 mg/mL, whereas the concentrations of Pf15-20 and Pf41-60 were relatively low, approximately 0.4 mg/mL and 1 mg/mL, respectively. For P4 and P5, the ratios of Pf15-20:Pf1-8 and Pf41-60:Pf1-8 were identical (0.1 and 0.3, respectively). Finally, for both P4 and P5, the relative proportions of the gelling peptides were much higher, at around 54%, which implied that the solutions contained less than 50% of non-gelling peptides.

## 3. Discussion

The aim of this study was to evaluate the influence of specific peptide species that are generated after tryptic hydrolysis of β-lactoglobulin on the purification and gelation capacity of Pf1-8. Our results show that the maximum Pf1-8 purity was close to 40% at the end of the production process. Despite the relative low purity of Pf1-8, gelation occurred, but it was largely dependent on the number of wash steps and peptide concentration. These results demonstrated that Pf1-8 was not the only major peptide involved in the gelation capacity of the recovered peptide pellet fractions. Other β-Lg peptides, namely Pf15-20 and Pf41-60, appeared to interact with Pf1-8 and participate in the gelation process.

### 3.1. Composition of β-Lactoglobulin Tryptic Hydrolysate during Filtration Processes

Our results showed the similarities between the peptide profiles of the initial β-lactoglobulin tryptic hydrolysates and the fractions that were obtained after filtration experiments (UFr and ROr as well as UFp) (Figure 1). However, variations on the relative amounts of the peptides were observed. These peptide profiles were mainly related to the molecular weight (MW) of the peptides as well as UF membrane selectivity. Indeed, all of the peptides had an MW lower than the molecular weight cut off (MWCO) of the membrane (10 kDa), which confirmed that pore size was the main factor that induced peptide transmission. The UF membrane may also contribute to the rejection of peptides in the retentate due to a repulsion phenomenon between the electronegative membrane material and peptides that were negatively charged at the pH of hydrolysate (pH 8.0) [30,31,32,33]. Protein–peptide and peptide–peptide interactions could also explain the presence of low MW peptides in the retentate. Many studies have been published describing this peptide aggregation phenomenon during whey protein hydrolysis by a wide range of enzymes, including trypsin [5,6,17,21,23,24,26]. Moreover, α-lactalbumin is known to interact with hydrophobic peptides that are generated by the hydrolysis of β-lactoglobulin by trypsin [29,34], whereas peptides f130-135, f69-83, and f146-149 interact with β-lactoglobulin [35] via electrostatic and hydrophobic interactions [32].

### 3.2. Impact of the Wash Steps on Fractionation, Recovery and Purity of Pf1-8

No trend was observed regarding the impact of the first two wash steps on the hydrolysate fractionation, since pellets and supernatants had similar peptide profiles (Figure 2). The high concentration of peptides in the initial hydrolysate, the diversity of peptide species (positively and negatively charged) and the possible formation of complex peptide aggregates through electrostatic interactions [5,29,32,36,37] could explain the low efficiency of these first two wash steps. However, our results clearly showed that fractionation between hydrophobic/hydrophilic peptides that occurred from the third wash step, since the pellets were mainly composed of hydrophobic peptides. Some hydrophobic peptides that were not detected in the first three wash steps were found in the pellet recovered after the last wash step, probably due to their very low initial relative abundance. Some of these peptides could originate from the trypsin hydrolysis of α-lactalbumin, which forms aggregates, particularly with Pf1-8 [21,23,29,34,37,38].

The recovery and purity of Pf1-8 were also improved from the third wash step, on. However, in addition to Pf1-8, two other peptides, Pf15-20 and Pf41-60, which represent more than 50% of the total peptide profile composition, were also detected in P4 and P5. Table 1 shows the amino acid compositions, the molecular weights, and the pI of these two other peptides. Similar to Pf1-8, the relative proportions of Pf15-20 and the Pf41-60 mostly dependent on the number of wash steps (Table 2). The presence of these specific peptides in a high proportion in the pellets is probably correlated to their high hydrophobicity, but also to the formation of complexes through hydrophobic and electrostatic interactions [29]. In agreement with the literature, these three peptides could participate in both electrostatic and hydrophobic interactions, resulting in aggregates [39,40]. Pf15-20 is neutral at pH 7 and it is mostly composed of hydrophobic amino acids. Thus, its behavior in our fractions is related to its ability to have hydrophobic interactions with other peptides. In addition, a peptide with a similar amino acid sequence (DIQKVAGTWY) has been characterized as an aggregating peptide [41,42]. Conversely, Pf41-60 is negatively charged (−2) at pH 7. This peptide is composed of alternating hydrophilic and hydrophobic amino acids. Indeed, Pf41-60 could be involved in both hydrophobic and electrostatic interactions, particularly with Pf1-8, which is positively charged at pH 7. The characteristics of Pf1-8 suggest that it would be an excellent candidate for self-assembly [29,32,37,39]. Surprisingly, the purity of Pf1-8 after five wash steps was drastically lower (38%) than that obtained in previous studies (close to 90%) [25,26,28]. This difference could be because the hydrolysate concentration that was used for the first wash step was higher (15% *w*/*v*) than the one used by Pouliot et al. (5%) [25]. At this time, the existing information does not provide a better explanation for this difference.

### 3.3. Gelation Capacities of Peptide Fractions

Our results demonstrated that the gelation capacity of the different peptide fractions (Table 3) was dependent on both the peptide concentration and profile. Globally and, as expected, as the Pf1-8 concentration increased, the lower the amount of initial peptide material that is required to induce gelation (from 40 mg/mL for P1, P2, and P3, to 20 mg/mL for P4 and, finally, 10 mg/mL for P5). According to Guy et al. [26], Pf1-8 can form a hydrogel at concentrations greater than 2.5 mg/mL. However, because different peptide fractions did not reach this threshold (P1 from 5 mg/mL to 20 mg/mL; P2 and P3 from 5 mg/mL to 10 mg/mL; and, P4 and P5 at 5 mg/mL) (Table 4), no gelation occurred (Table 3). Gel formation was also observed for fraction P1 at high concentrations (40, 50, and 75 mg/mL). Surprisingly, this fraction had a very low relative percentage of Pf1-8 (close to 0.7 mg/mL). Consequently, these results demonstrate that Pf1-8 was not solely responsible for gel formation and that other peptides had gelling capacity [32]. For P1 fractions, the concentration of Pf15-20 and Pf41-60 were higher than that of Pf1-8. While these peptides were not previously identified as gelling peptides, they are known to form aggregates that could induce the formation of a gel [5,6,21,26]. Gel formation was observed for P2 and P3 at the same concentration of peptide material (20 mg/mL). Similar to P1, P2 fractions had very low amounts of Pf1-8 (0.16 mg/mL), but the gelation that occurred was probably due to the presence of high proportions of Pf15-20 and Pf41-60. Contrary to P2, the gelation of P3 was probably due to its content of Pf1-8 (3.08 mg/mL), which exceeds the gelation threshold of 2.5 mg/mL for this peptide. These results showed that gel formation is only partly dependent on Pf1-8 concentration. as we cannot rule out the potential role of both Pf15-20 and Pf41-60 in the gelation mechanism.

## 4. Materials and Methods

### 4.1. Preparation of Whey Protein Solutions

WPI (BiPRO) was purchased from Agropur Ingredients (Le Sueur, MN, USA). According to the manufacturer, the proximate composition of WPI was 97.5% protein, 0.6% fat, and 1.8% ash on a dry basis. Three independent batches of protein solutions (9.80% (*w*/*v*)) were prepared by the solubilization of 6.65 kg of WPI in 68.0 kg of deionized water. Whey protein solutions were stirred overnight at 4 °C until being used for tryptic hydrolysis.

### 4.2. Production of Tryptic Hydrolysate of Whey Proteins

The tryptic hydrolysate was prepared according to the protocol of Pouliot et al. [25]. Briefly, trypsin solution (Trypsin VI) from Neova technologies (Abbotsford, BC, Canada) was solubilized at 10% (*w*/*v*) in HCl (0.001 N) and it was added to whey protein solutions at an enzyme-to-substrate (E/S) ratio of 1/700. Hydrolysis was carried out at 38 °C, pH 8 using the pH-stat method. When a degree of hydrolysis (DH) of 5.6% was reached, the enzymatic reaction was stopped by separating the reaction products (tryptic peptides) from the enzyme and substrate (non-hydrolyzed proteins) by UF (polyethersulfone membrane with MWCO of 10 kDa) while using a GEA Niro system from GEA Niro Inc. (Düsseldorf, Germany). UF was performed for 100 min at 35.5 °C under a constant transmembrane pressure (TMP) of 4.5 bar. During UF, a discontinuous diafiltration (DF) was carried out. The UFr was diluted with 1 diavolume (*DV*, Equation (1)) twice and the diluted UFr was concentrated to a final volume concentration factor (VCF) of 10×.

(1) $$DV=\frac{V_{w}}{V_{UFr}}$$

where $V_{w}$ corresponds to the volume (L) of water added to retentate and $V_{UFr}$ is the volume (L) of UFr.

After UF, the three batches of tryptic hydrolysate of whey proteins (UFp) were stored at 4 °C until concentration by RO. The samples of hydrolysates produced before UF (H), as well as after UFp and UFr from each batch, were also collected, freeze-dried, and stored at −18 °C until analysis.

### 4.3. Production, Concentration and Purification of Tryptic Peptides of Whey Proteins with Gelation Properties

**Figure 3:**
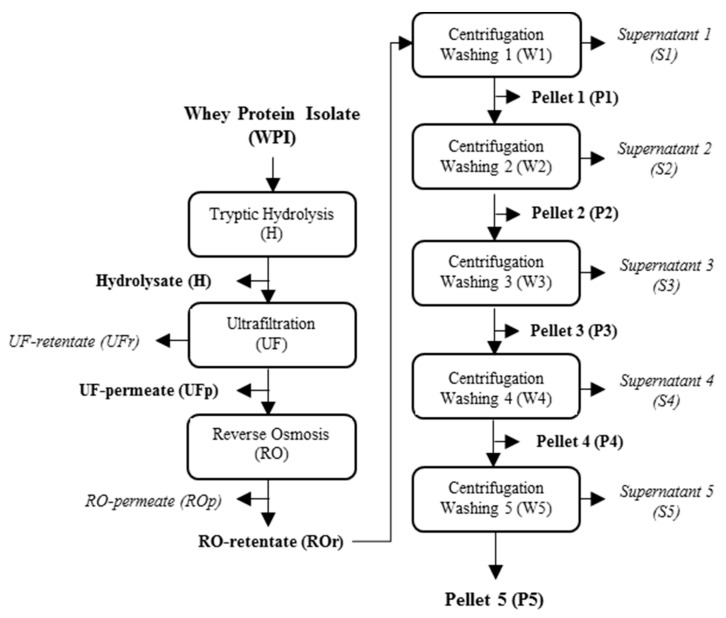
Flow chart of the process for the production and purification of Pf1-8.

Figure 3 shows a flowchart of the process for the production and purification of Pf1-8. The three batches of tryptic hydrolysates of whey protein (UFp) were concentrated by RO at 22.5 °C under a constant TMP of 20.5 bar for 2 h, as described by Pouliot et al. [25]. A VCF of 6× was reached at the end of RO. After concentration by RO, the three ROr were freeze-dried and then solubilized in Milli-Q water at neutral pH, at a concentration of 15% (*w*/*v*). Five wash steps were performed by centrifugation to purify Pf1-8, as described by Pouliot et al. [25]. After each wash the peptide yield was determined according to Equation (2).

(2) $$Y\left(\%\right)=\frac{W_{pellet}}{W_{ROr}}\times 100$$

where $W_{pellet}$ corresponds to the total weight of pellet that was obtained after centrifugation and lyophilization, and $W_{ROr}$ is the weight of ROr dissolved in milliQ water for the centrifugation.

Finally, samples of ROr, pellets (P1 to P5) and supernatants (S1 to S5) were collected and then stored at −18 °C until analysis.

### 4.4. Peptide Composition

#### 4.4.1. Protein Content

The total nitrogen content of H, UFp, UFr, ROr, and P1 to P5 samples was determined in duplicate using the Dumas method with a LECO FP-528 nitrogen analyzer (St. Joseph, MI, USA). The nitrogen-to protein conversion factor of 6.38.

#### 4.4.2. Peptide Characterization

The peptide profiles of H, UFp, UFr, ROr, P1 to P5, and S1 to S5 samples were determined by RP-HPLC using an HPLC Agilent system that was equipped with a variable UV-visible detector operating at 214 nm (series 1100, Agilent Technologies) (Palo Alto, CA, USA), as described by Pouliot et al. [25]. For each chromatogram (Figure 1 and Figure 2), the relative percentage of each peak numbered from 1 to 25 was calculated by dividing the area under the curve for the peak corresponding to one peptide/protein species by the total area under the curve for all peptides/proteins, including unidentified peptides [43]. Analyses were performed at 45 °C while using a linear gradient of solvent B over 70 min. Solvent A was composed of 0.1% (*v*/*v*) trifluoroacetic acid (TFA) in water and solvent B was composed of 90% (*v*/*v*) acetonitrile, 10% (*v*/*v*) water, and 0.1% TFA. All of the freeze-dried pellets (P1 to P5) were rehydrated to 5% protein (*w*/*v*) in Milli-Q water and then diluted to a ratio of 1/6 with solvent A. S1 were diluted 1/10 in a stock solution that was composed of Milli-Q water:solvent A at a ratio of 1:5. The other supernatants (S2 to S5) were injected without dilution into the HPLC system.

### 4.5. Gelation Properties of Whey Peptide Fractions

Gelation experiments were conducted on pellets (P1 to P5) that were recovered after each of the five wash steps. All of the freeze-dried samples were first solubilized in Milli-Q water at pH 2 and then stirred for 30 min. Different concentrations (gelation threshold concentrations were determined by preliminary tests (the results not shown)) of peptide fractions recovered in washed pellets were tested (75 to 5 mg/mL for P1, 20 to 5 mg/mL for P2 and P3, and 10 to 5 mg/mL for P4 and P5). The pH of each solution was adjusted to 11 with 2N NaOH [26] and the solutions were stored at 4 °C to induce gel formation. Gelation was confirmed if the gel was not deformed when the tube was inverted.

### 4.6. Statistical Analysis

The production was performed in triplicate. The relative proportions and concentrations of peptide f1-8, f15-20 and f41-60 results were expressed as mean ± standard deviation (SD) and were analysed by mean comparison using the Student’s *t*-test (Tukey) (SAS software, version 13, SAS Institute Inc., Cary, NC, USA). For all statistical analyses, a *p*-value of <0.05 was considered statistically significant.

## 5. Conclusions

This study examined the impact of wash steps on the purification and gelation capacity of Pf1-8. As expected, the purity of Pf1-8 correlated with the number of wash steps. Two other peptides (Pf15-20 and Pf41-60) were identified as the main components during Pf1-8 fractionation and purification. While gel formation was observed when the Pf1-8 concentration was higher than its gelation threshold, gelation also occurred at very low relative proportions of Pf1-8 in the presence of other peptides (Pf15-20 and Pf41-60). The potential peptide–peptide interactions between Pf1-8, Pf15-20 and Pf41-60 could explain these observations. Because both temperature and pH are known to influence the hydrophobic and electrostatic interactions involved in peptide-peptide interactions, those parameters are currently under study to improve the efficiency of the Pf1-8 wash steps. The study of the secondary structure of peptide f15-20 and f41-60 as well as a characterization of the gels using rheological studies and microscopy could be considered in order to deepen the understanding of the gelation process.

## Figures and Tables

**Table 1:** Amino acid composition and characteristics of peptides identified in the tryptic hydrolysate and the peptide fractions obtained during filtration (ultrafiltration (UF) and reverse osmosis (RO)) and wash steps.

Peak ^a^	Peptide Sequence	Amino Acid Composition	MW ^b^ (g/mol)	pI ^c^	Hydrophobicity ^d^ (kcal/Residue)
1	139–141	ALK	331.2	8.80	1.55
2	146–148	HIR	424.3	9.76	1.23
3	136–138	FDK	409.2	5.84	1.38
4	41–42	VY	280.2	5.49	2.28
5	71–75	IIAEK	573.4	6.00	1.63
6	33–40	DAQSAPLR	856.5	5.84	0.91
7	84–91	IDALNENK	916.5	4.37	0.95
8	83–91	KIDALNENK	915.5	6.07	1.18
9	33–39	DAQSAPL	700.3	3.80	0.93
10	9–14	GLDIQK	673.4	5.84	1.14
11	125–135	TPEVDDEALEK	1245.6	3.83	0.85
12	1–8	LIVTQTMK	933.5	8.75	1.34
13	142–148	ALPMHIR	837.5	9.80	1.54
14	92–101	VLVLDTDYKK	1193.2	5.93	1.45
15	125–138	TPEVDDEALEKFDK	1635.4	4.02	0.97
16	15–20	VAGTWY	694.5	5.49	1.46
17	92–100	VLVLDTDYK	1065.6	4.21	1.44
18	61–70 + 149–162	WENGECAQKK + LSFNPTQLEEQCHI	2928.4	4.57	0.89
19	76–82	TLIPAVFK	774.4	8.41	1.88
20	78–82	IPAVF	545.3	5.52	2.13
21	43–60	VEELKPTPEGDLEILLQK	2052.6	4.25	1.13
22	21–40	SLAMAASDISLLDAQSAPLR	2029.7	4.21	1.05
23	41–60	VYVEELKPTPEGDLEILLQK	2323.3	4.25	1.24
24	21–32	SLAMAASDISLL	1190.7	3.80	1.14
25	α-lactalbumin	-	14,176.2	-	-

^a^ Peak numbers from Figure 1 and Figure 2. ^b^ Molecular weights. ^c^ Calculated by Expasy Molecular biology. ^d^ Calculated according to the method of Bigelow [25]).

**Table 2:** Relative proportions and concentrations of Pf1-8, Pf15-20, and Pf41-60 in pellets (P1 to P5) and supernatants (S1 to S5) recovered after each wash step.

	Pf1-8		Pf15-20		Pf41-60	
	Relative Proportion (%)	Concentration (mg/mL)	Relative Proportion (%)	Concentration (mg/mL)	Relative Proportion (%)	Concentration (mg/mL)
P1	0.85 ± 0.09 ^a^	0.07 ± 0.00 ^a^	15.1 ± 0.06 ^a^	1.36 ± 0.01 ^a^	9.21 ± 0.23 ^a^	0.83 ± 0.01 ^a^
S1	0.72 ± 0.00 ^a^	ND *	14.1 ± 0.00 ^a^	ND *	9.48 ± 0.00 ^a^	ND *
P2	0.81 ± 0.15 ^a^	0.07 ± 0.00 ^a^	15.1 ± 1.87 ^a^	1.34 ± 0.01 ^a^	9.21 ± 0.48 ^a^	0.82 ± 0.01 ^a^
S2	0.88 ± 0.05 ^a^	ND *	12.6 ± 1.76 ^a,b^	ND *	8.63 ± 0.48 ^a^	ND *
P3	15.4 ± 4.43 ^b^	1.30 ± 0.01 ^b^	15.6 ± 0.04 ^a^	1.41 ± 0.01 ^b^	5.80 ± 5.24 ^a^	0.52 ± 0.00 ^b^
S3	0.79 ± 0.16 ^a^	ND *	20.2 ± 0.98 ^a,c^	ND *	6.44 ± 5.58 ^a^	ND *
P4	35.9 ± 3.15 ^c^	3.29 ± 0.02 ^c^	4.15 ± 0.37 ^b^	0.38 ± 0.00 ^c^	11.1 ± 0.50 ^a^	1.02 ± 0.01 ^c^
S4	-	ND *	27.1 ± 3.49 ^c^	ND *	13.4 ± 0.73 ^a^	ND *
P5	38.9 ± 4.09 ^c^	3.57 ± 0.06 ^d^	3.99 ± 1.61 ^b^	0.37 ± 0.01 ^c^	10.8 ± 1.39 ^a^	0.99 ± 0.02 ^c^
S5	-	ND *	25.5 ± 5.24 ^c^	ND *	-	ND *

Proportions (%) and concentrations (mg/mL) are means ± SD of duplicate measurements. * Not Determined (due to the very low peptide concentration in the supernatants). Means within the same column with different subscript letters (a–d) are significantly different (Tukey test, α = 0.05).

**Table 3:** Gelling ability of pellets solubilized at different concentrations and adjusted to pH 11.

	Peptide Concentration (mg/mL)					
	5	10	20	40	50	75
P1	−	−	−	+	+	+
P2	−	−	+	ND	ND	ND
P3	−	−	+	ND	ND	ND
P4	−	+	ND	ND	ND	ND
P5	−	+	ND	ND	ND	ND

(+) indicates gelling while (−) indicates the absence of gel formation. ND: Gelation was not tested for these concentrations (see preliminary results explained in Section 4).

**Table 4:** Concentrations of peptides Pf1-8, Pf15-20, and Pf41-60 in pellets (P1 to P5) for each solution assessed for gelling ability.

Pellet Fraction Concentration (mg/mL)	Pf1-8 Concentration (mg/mL)	Pf15-20 Concentration (mg/mL)	Pf41-60 Concentration (mg/mL)
P1 5	0.04 ± 0.01 ^a^	0.76 ± 0.00 ^a^	0.46 ± 0.01 ^a^
P1 10	0.09 ± 0.01 ^a^	1.51 ± 0.01 ^b^	0.92 ± 0.02 ^a,b^
P1 20	0.17 ± 0.02 ^a^	3.03 ± 0.01 ^c^	1.84 ± 0.05 ^b^
P1 40 *	0.34 ± 0.04 ^a^	6.05 ±0.02 ^e^	3.69 ± 0.09 ^c^
P1 50 *	0.43 ± 0.05 ^a,b^	7.56 ± 0.03 ^f^	4.61 ± 0.11 ^c^
P1 75 *	0.64 ± 0.07 ^a,b,c^	11.3 ± 0.04 ^g^	6.91 ± 0.17 ^d^
P2 5	0.04 ± 0.01 ^a^	0.76 ± 0.09 ^a^	0.46 ± 0.02 ^a^
P2 10	0.08 ± 0.02 ^a^	1.51 ± 0.19 ^b^	0.92 ± 0.05 ^a,b^
P2 20 *	0.16 ± 0.03 ^a^	3.02 ± 0.37 ^c^	1.84 ± 0.1 ^b^
P3 5	0.77 ± 0.22 ^a,b,c^	0.78 ± 0.00 ^a^	0.29 ± 0.26 ^a^
P3 10	1.54 ± 0.44 ^b,c,d^	1.56 ± 0.00 ^b^	0.58 ± 0.52 ^a^
P3 20 *	3.08 ± 0.89 ^e,f^	3.12 ± 0.01 ^c^	1.16 ± 1.05 ^a,b^
P4 5	1.79 ± 0.16 ^c,d^	0.21 ± 0.02 ^d^	0.55 ± 0.03 ^a^
P4 10 *	3.59 ± 0.32 ^f^	0.42 ± 0.04 ^a,d^	1.11 ± 0.05 ^a,b^
P5 5	1.94 ± 0.20 ^d,e^	0.20 ± 0.08 ^d^	0.54 ± 0.07 ^a^
P5 10 *	3.88 ± 0.41 ^f^	0.40 ± 0.16 ^a,d^	1.08 ± 0.14 ^a,b^

* sample concentration at which a gel was obtained. Concentrations (mg/mL) are means ± SD of duplicate measurements. Means within the same column with different subscript letters (a–g) are significantly different (Tukey test, α = 0.05).

## Data Availability

The data presented in this study are available in the article.

## Supplementary Materials

The following are available online. Figure S1: Gelling ability of P1 (a), P2 (b), P3 (c), P4 (d) and P5 (e) solubilized at different concentrations and adjusted to pH 11.

## Sample Availability

Samples of the compounds are not available from the authors.
