# Supplementary material for: Occurrence of Peptide-Peptide Interactions during the Purification of Self-Assembling Peptide f1-8 from a β-Lactoglobulin Tryptic Hydrolysate
Source: Molecules. 2021 Mar 6;26(5):1432. doi: 10.3390/molecules26051432 (PMC7961507; doi:10.3390/molecules26051432)
Supplement: Supplementary file 1 [file molecules-26-01432-s001.pdf]

Supplementary Materials:

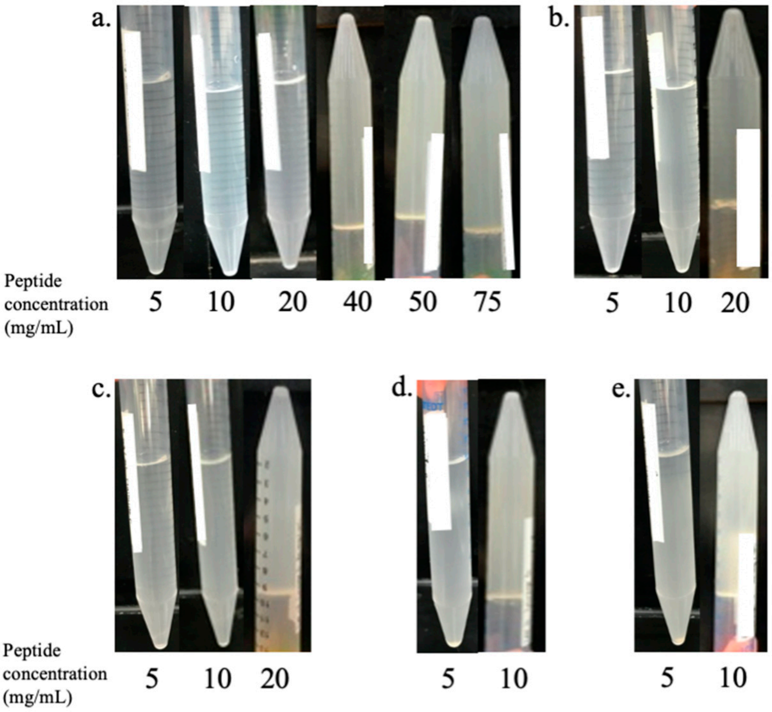

Supplementary material 1. Gelling ability of P1 (a), P2 (b), P3 (c), P4 (d) and P5 (e) solubilized at different concentrations and adjusted to pH 11.
